# Comparison of three port placement strategies for robot-assisted laparoscopic lich-gregoir direct nipple ureteral extravesical reimplantation in pediatric primary obstructive megaureter: multi-ports, single-port-plus-one, single-port

**DOI:** 10.3389/fped.2025.1691765

**Published:** 2025-11-06

**Authors:** Yuru Zhang, Jingjing Lu, Shan Lin, Shaohua He, Di Xu, Jianglong Chen

**Affiliations:** 1 Shengli Clinical Medical College, Fujian Medical University, Fuzhou, China; 2 Department of Pediatric Surgery, Fuzhou University Affiliated Provincial Hospital, Fuzhou, China

**Keywords:** robot-assisted laparoscopic, primary obstructive megaureter, Lich-Gregoir direct nipple, multi-ports, single-port-plus-one, single-port

## Abstract

**Objective:**

This study aimed to evaluate the efficacy, safety, and cosmetic outcomes of three robot-assisted laparoscopic techniques for treating pediatric primary obstructive megaureter (POM): robot-assisted laparoscopic multi-ports (RLMG), robot-assisted laparoscopic single-port-plus-one (RLSPG), and robot-assisted laparoscopic single-port (RLSG).

**Materials and methods:**

A retrospective analysis included 30 pediatric POM patients (December 2022–December 2024) undergoing Da Vinci Xi robotic Lich-Gregoir ureteral reimplantation. Patients were categorized into RLMG (*n* = 13), RLSPG (*n* = 10), and RLSG (*n* = 7) groups based on incision methods. Preoperative assessments, Intraoperative parameters, postoperative outcomes, and hydronephrosis metrics were analyzed using SPSS 21.0, with significance set at *P* < 0.05.

**Results:**

There were no significant differences in baseline demographic characteristics. There were significant inter group differences in the distribution of surgical side (*P* = 0.005). In terms of total surgical time, the RLMG group was significantly shorter than the RLSPG and RLSG (*P* = 0.02). There was no significant difference in ureteral reimplantation time among the three groups (*P* = 0.85), but the ratio of ureteral reimplantation time to total surgical time in the RLSPG and RLSG was significantly lower than that in the RLMG (*P* < 0.001). The Stony Brook Scar Evaluation Scale (SBSES) score showed that the RLSG had significantly higher scores than the RLMG (*P* = 0.009) and RLSPG (*P* = 0.244). After surgery, only 2 cases of RLMG, 3 cases of RLSPG, and 2 cases of RLSG developed urinary tract infections, all of which were relieved through conservative treatment without recurrence. In terms of follow-up time, the RLMG had the longest median follow-up time, with significant differences between groups (*P* < 0.001). The relief rate of obstruction in all three groups was 100%. Postoperative renal hydronephrosis parameters were significantly improved compared to preoperative levels (*P* < 0.001).

**Conclusion:**

Robot-assisted laparoscopic ureteral reimplantation is a safe and effective treatment for pediatric POM. The single-port approach achieves superior cosmesis, whereas the multi-ports technique affords the shortest operative time. The single-port-plus-one offers a balanced intermediate option, enabling surgeons to optimize outcomes based on patient and procedural needs.

## Introduction

The total incidence rate of POM is about 1:1,500–1:2,000, which is one of the main causes of renal dysfunction in children (1). The British Society of Pediatric Urology believes that from 30 weeks of pregnancy onwards, a diameter of the extravesical ureter greater than 7 mm can be diagnosed as megaureter (2, 3). Relieving urinary tract obstruction and protecting renal function are important treatment goals for congenital megaureter. Most children can alleviate disease progression through non-surgical treatment, but there are still some children who require surgical intervention (4).

Ureteral reimplantation is the standard surgery for the treatment of primary obstructive megaureter. According to the surgical approach, it can be divided into intravesical and extravesical methods (5). The Lich Gregoir procedure is a classic procedure for extravesical ureteral reimplantation. In the early stages, open surgery was the main method of this procedure. With the rapid development of minimally invasive surgical technology and the implementation of minimally invasive concepts, laparoscopy and robot assisted laparoscopy have been widely used in this procedure (6, 7). The advantages of robot 3D visualization, tremor filtering, and motion scaling can help surgeons achieve finer sutures and more precise operations, which have potential advantages in the reconstruction of structural deformities in children. Currently, they are most widely used in pediatric urology. Since 2020, our department has carried out the first robotic laparoscopic surgery for children.

In order to reduce the harm to children and minimize the size and number of incisions, we have continuously improved our technology. Our robot assisted laparoscopic technology has undergone a transformation from multiple ports to single-port-plus-one (8, 9). With the accumulation of previous experience, we have now achieved the application of single-port technology in the treatment of common pediatric urinary system deformities, including POM. The impact of different perforation methods on the appearance and efficacy of postoperative wounds in children is not yet clear. In this study, we evaluated the short-term and long-term effects of three perforation methods on POM children by collecting and comparing clinical data from three groups of children.

## Methods

### Patients and design

This study retrospectively analyzed the case data of 30 pediatric patients who underwent surgical treatment for POM in our department from December 2022 to December 2024. The decision for surgical intervention was based on one or more of the following criteria: (1) The presence of clinical symptoms such as recurrent urinary tract infections or flank/abdominal pain; (2) Radiological evidence of progressive hydronephrosis or severe ureteral tortuosity on MRU; (3) Impaired renal function on diuresis renography, defined as a differential renal function (DRF) of less than 40% on the affected side, or a progressive decline of >5% in DRF.

They were divided into three groups: robot-assisted laparoscopic multi-ports group (RLMG, *n* = 13), robot-assisted laparoscopic single-port-plus-one group (RLSPG, *n* = 10), and robot-assisted single-port group (RLSG, *n* = 7). Inclusion criteria: Children who underwent the first da Vinci robot assisted laparoscopic Lich Gregoir ureteral reimplantation surgery in our department; Complete clinical data; Follow up after surgery for more than 3 months. Exclusion criteria: Secondary obstructive megaureter caused by neurogenic bladder, ureteral protrusion, posterior urethral valve, etc. All patients were divided into porous group, single well group, and single well plus one group according to the surgical perforation method. This study has been approved by the Fujian Provincial Hospital Institutional Review Committee (Approval Number: K2022-07-008) and has obtained informed consent from all families of the included patients.

All patients completed magnetic resonance urography (MRU), 99m Tc-mercaptoacetyltriglycine (MAG3), 99Tcm-dimercaptosuccinic (DMSA) and voiding cystourethrogram (VCUG) before surgery.

### Surgical approaches

All surgeries in this study were performed by the same same surgeon and surgical team using the Da Vinci Xi robotic surgical system (Da Vinci, Mountain View, CA, USA).The surgical steps of the three groups of patients are similar, mainly due to the different perforation methods.

#### RLMG

**Figure 1:**
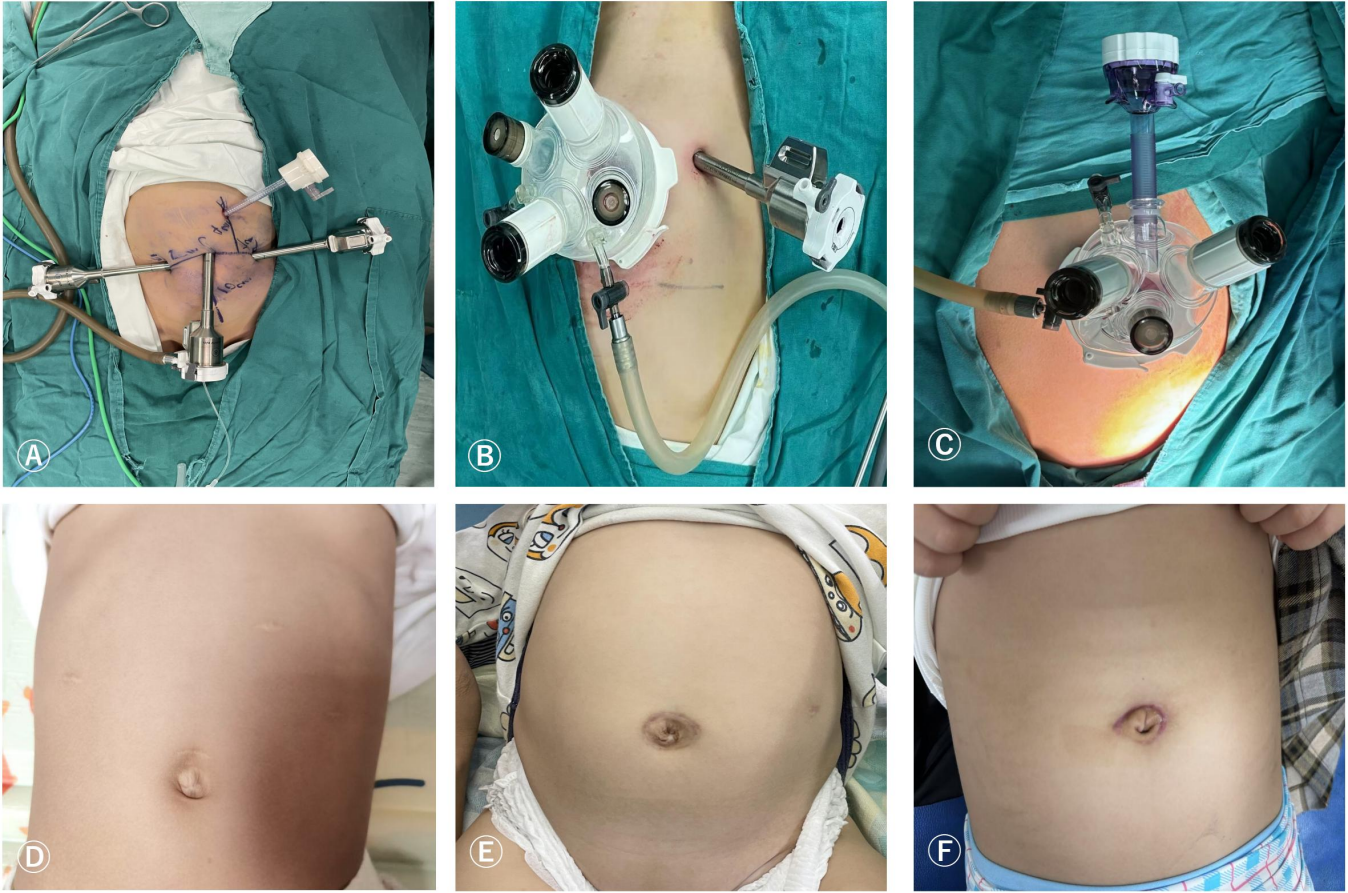
Surgical port placement and postoperative wound healing of RLMG **(A,D)**, RLSPG **(B,E)**, and RLSG **(C,F)**.

Take an approximately 8 mm incision on the upper edge of the navel, place an 8.0 mm 3D camera trocar connected to the third robotic arm, and use this incision as the center. Take another approximately 8 mm incision at each end about 6 cm away from it, and place the 8.0 mm operating trocar connected to the second and fourth robotic arms respectively. Place another 5 mm assistant operating trocar above the connection between the two robotic arms on the surgical side, 4 cm away from the second or fourth operating arm (Figure 1A).

#### RLSPG

Take a 3.5 cm arc-shaped incision around the left edge of the navel and place a single hole four channel puncture device, usually consisting of 2 1 cm channels and 2 5 mm channels. The four channels will be used to place an 8.0 mm 3D camera trocar connected to the third robotic arm, an operation trocar connected to the fourth robotic arm, and an assistant assisted operation trocar. Perform another 8 mm operation to connect the trocar to the 2nd robotic arm at a distance of 6 cm from the 3D camera trocar on the left or right abdomen according to the surgical location (Figure 1B).

#### RLSG

Take an arc-shaped incision around the left edge of the navel, place a single hole device, and we will modify it into 3 1 cm channels and 1 5 mm channel. Three 1 cm channels are used to insert three 8 mm robot operated trocar and lens trocar, and the remaining one 5 mm channel is used as an assistant auxiliary channel. Two operation trocars are placed tightly on both sides of the single port base, and the lens sheath is placed between the two operation trocars, floating in the single port (Figure 1C).

After endotracheal intubation combined with general anesthesia, the patient is placed in a supine position with the head low and feet high at 30°. Underneath the warm blanket, use sponge pads to cushion the lower limbs under stress, and let both upper limbs naturally sag. After routine disinfection and towel laying, a catheter is left in place. The end of the catheter is connected to a three-way switch, with one end connected to a drainage bag and the other end connected to a 50 ml syringe.

**Figure 2:**
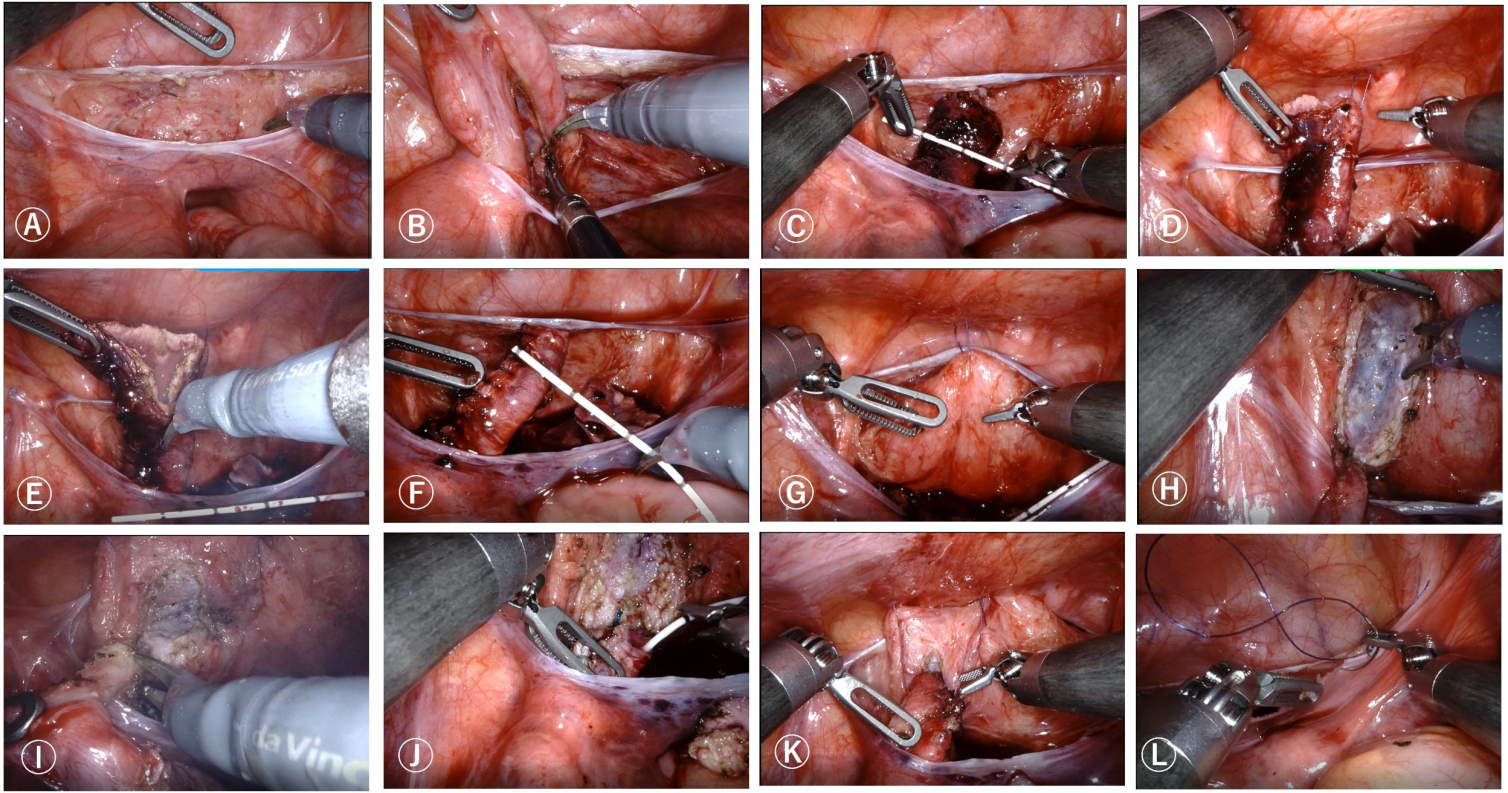
Robot-assisted laparoscopic Lich-Gregoir direct nipple ureteral extravesical reimplantation surgical procedures. **(A)** Open the outer peritoneal fold of the bladder. **(B)** Bluntly separate surrounding ureteral tissue. **(C)** Measure the width of the dilated ureter. **(D,E)** Remove the narrow segment of the ureter, suspend and cut the dilated ureter. **(F)** Remeasure the width of the ureter. **(G)** Suspend the bladder to the abdominal wall. **(H)** Open the bladder serosa layer. **(I)**. Anastomosis of the ureter and bladder. **(J)**. Insertion of a double-J tube. **(K)**. Form a ureteral tunnel, embedding and suturing the ureter between the bladder muscle layers. **(L)**. Suture the peritoneum.

Each group takes the above-mentioned incision method and establishes pneumoperitoneum (pressure 8–12 mmHg, flow rate 3–5 L/min). Connect each operating hole to the robotic arm, enter the abdominal cavity, open the outer peritoneal fold of the bladder (Figure 2A), directly identify the free ureter on the anterior outer side of the vas deferens or uterine artery, bluntly separate the surrounding structure, separate the ureter downwards to the bladder connection (Figure 2B), measure the width of the dilated ureter (Figure 2C), disconnect the ureter near the bladder, remove the narrow segment of the ureter, suspend and cut the dilated ureter (Figures 2D,E), suture the ureter to form and re measure the width of the ureter (Figure 2F), insert a 4-0 line above the pubic symphysis outside the abdominal cavity, suspend the bladder to the abdominal wall (Figure 2G), insert sterile physiological saline through a catheter, fill the bladder, measure the length of the bladder plasma muscle layer incision and mark it, generally 4–5 times the width of the ureter after cutting and forming, open the bladder serosa layer, and at a distance of 1.5 cm from the end of the ureter (Figure 2H). Using 6-0 Polydioxanone Suture at the 6 o'clock and 12 o'clock positions, Intermittent suturing of the ureteral seromuscular layer and the entire bladder wall, followed by 4 stitches in each quadrant to complete direct nipple implantation at the end of the ureter and insertion of a double-J tube (Figures 2I,J). From bottom to top, intermittently suture the ureter and detrusor muscle to form a ureteral tunnel, embedding and suturing the ureter between the bladder muscle layers (Figure 2K).Cut off the suspension traction line, clean the wound with warm physiological saline, wash away the intra-abdominal fluid, confirm no active bleeding or leakage, and suture the peritoneum (Figure 2L). Evacuate the pneumoperitoneum and all robotic arm operating instruments, close the pneumoperitoneum, and suture the incision layer by layer.

### Patient postoperative management

Our postoperative management follows a conservative protocol to ensure optimal healing in children. A liquid diet is initiated only after the confirmation of bowel recovery to lower the risk of postoperative ileus. When eating is normal and there are no complications such as anastomotic leakage or incision infection, the patient can be discharged. Remove the catheter 14 days after surgery. The prolonged catheterization aims to maintain a low-pressure bladder environment, reducing tension on the fresh ureterovesical anastomosis and minimizing the risk of urinary leakage. The double-J stent is removed after 1–2 months to allow for complete healing of the anastomosis before its removal, thereby preventing late stricture formation. If there is unexplained fever after surgery, it is recommended to have a urinary system color doppler ultrasound and urine routine examination.It is recommended to have a ultrasound of the urinary system at 1, 3 months after surgery, and MRU, MAG-3, DMSA and VCUG at 6–12 months after surgery.

### Data collection

The collected data includes: preoperative parameters [age, gender, height, weight, surgical side, preoperative anteroposterior diameter (APD) of renal pelvis, society for fetal urology (SFU) grade, ureteral diameter, differential renal function (DRF)%, reflux level], intraoperative and postoperative parameters (total operative time, blood loss, fasting time, retention time of catheter, hospitalization and incidence of complication), follow-up parameters (follow-up time, outcomes and parents' satisfaction scores to the surgical scar according to SBSES).

### Statistical analysis

This study used SPSS Statistics version 21.0 (IBM Corp., New York City, NY, USA) to process the data. Metric data that follows a normal distribution are represented by mean ± standard deviation (SD), while metric data that does not follow a normal distribution are represented by median (range). Use Student's t-test to compare continuous variables and chi square test to compare categorical variables. A value of *P* < 0.05 is considered statistically significant.

## Results

No significant differences were observed among the three groups in demographic characteristics, including age, gender, height, weight, or initial symptoms (*P* > 0.05). All groups had children under the age of 1, with 3 child in RLMG, 5child RLSPG, and 3 child in RLSG, with no significant intergroup difference (*P* = 0.448). A significant difference was noted in the distribution of surgical sides (*P* = 0.005), with the majority of left-sided POM cases being in the RLSPG (9/10) and RLSG (6/7) groups, while the RLMG group had a predominance of right-sided cases (9/13). The majority of POM cases were classified as “obstruction without refluxing”, with 8 cases in RLMG, 8 case in RLSPG, and 6 case in RLSG, with no significant intergroup difference (*P* = 0.51). Among the reflux levels indicated by VCUG examination showed higher prevalence of grades IV–V across groups, though without statistical significance (*P* = 0.258) (Table 1).

**Table 1:** Preoperative parameters of the patient.

Parameters	RLMG (*n* = 13)	RLSPG (*n* = 10)	RLSG (*n* = 7)	*P*
Age (months)	36 (4.72)	18 (8.132)	24 (1.240)	0.947
<1 year	3	5	3	0.448
≥1 year	10	5	4	
Gender				
Male	8	8	4	0.617
Female	5	2	3	
Height (cm)	98 (64.126)	85 (71.148)	89 (54.175)	0.741
Weight (kg)	16 (8.26)	14 (9.42)	3 (5.65)	0.934
Surgical side				
Left	4	9	6	0.005
Right	9	1	1	
Initial symptoms				
Yes	1	2	3	0.801
No	12	8	4	
The type of POM				
Obstruction with refluxing	5	2	1	0.51
Obstruction without refluxing	8	8	6	
Reflux level^a^				
I	2	0	0	0.258
II	0	0	0	
III	1	0	1	
IV	3	1	0	
V	3	1	0	

a According to the grading criteria of voiding cystourethrography (VCUG) used by the International Reflux Research Group in 1985.

There was a significant difference in the total surgical time among the groups (*P* = 0.02), with RLMG group (154.38 ± 10.97 min) being significantly shorter than RLSPG group (167.10 ± 11.20 min) and RLSP group (169.57 ± 16.97 min), but there was no significant difference between RLSPG group and RLSP group (*P* = 0.721). However, the time for ureteral reimplantation in the abdominal cavity of the three groups was roughly the same (*P* = 0.85), with RLMG group taking 105.77 ± 10.59 min and RLSPG group taking 104.80 ± 8.75 min, and RLSG taking 107.71 ± 11.74 min. There was a significant difference in the ratio of ureteral reimplantation time to total surgical time among the three groups, with RLMG accounting for a significantly higher ratio than RLSPG and RLSG (*P* < 0.001). Blood loss, fasting time, catheter retention time, hospitalization days, postoperative complication rates (including urinary tract infections) showed no significant differences (*P* > 0.05). There were no other complications after surgery except for urinary tract infection. There were 2 cases in RLMG, 3 case in RLSPG, and 2 case in RLSG who developed urinary tract infection within 1 months after surgery. All cases were treated conservatively, and there was no recurrence after removing the double-J tube. There were significant differences in Surgical scar evaluation (SBSES scores) among the groups (*P* = 0.016), with RLSG group significantly higher than RLSG group (*P* = 0.009) and higher than RLSPG group, but the difference was not statistically significant (*P* = 0.244) (Table 2).

**Table 2:** Perioperative parameters of the patient.

Parameters	RLMG (*n* = 13)	RLSPG (*n* = 10)	RLSG (*n* = 7)	*P*
Total operative time (min)	154.38 ± 10.97	167.10 ± 11.20	169.57 ± 16.97	0.02
Ureteral reimplantation time (min)	105.77 ± 10.59	104.80 ± 8.75	107.71 ± 11.74	0.85
Ratio%	68.44 ± 3.40%	62.69 ± 2.45%	63.51 ± 2.34%	<0.001
Blood loss (ml)	18.77 ± 3.40	16.10 ± 2.19	16.43 ± 1.78	0.149
Fasting time(h)	19.62 ± 2.81	18.30 ± 1.95	19 ± 2.52	0.464
Gross hematuria time(days)	2.23 ± 1.09	2.00 ± 1.15	2.29 ± 1.11	0.843
Retention time of catheter (days)	14 (11,15)	14 (9,15)	14 (13,15)	0.637
Hospitalization (days)	7 (6,7)	7 (6,10)	7 (7,7)	0.203
Complication	2	3	1	0.724
Anastomotic stenosis	0	0	0	
Urinary tract infection	2	3	1	
SBSES scores of surgical scar	5.08 ± 1.38	4.00 ± 1.25	3.29 ± 1.11	0.016

SBSES, stony brook scar evaluation scale.

The RLMG had the earliest implementation time and the longest follow-up time, the median follow-up duration 12(6.36) months was longest, with significant intergroup variation (*P* < 0.001). As summarized in Table 3, APD, ureteral diameter, SFU grade and difference in DRF showed a significant reduction at the last follow-up compared to preoperative values in all three groups (*P* < 0.001), confirming the relief rate of obstruction in all three groups was 100%. The preoperative anteroposterior diameter (APD) and maximum ureteral diameter are as follows: for the RLMG group, 39.42 ± 13.64 mm and 17.73 ± 4.44 mm; for the RLSPG group, 22.37 ± 7.23 mm and 20.10 ± 3.64 mm; for the RLSG group, 30.10 ± 13.71 mm and 17.60 ± 3.06 mm. The preoperative SFU grading was mainly grade IV (7 cases in RLMG group, 5 cases in RLSPG group, and 3 cases in RLSG group), and the proportion of grade I-II significantly increased during postoperative follow-up (*P* = 0.048). In children with preoperative POM type of obstruction with refluxing, most reflux disappeared after surgery. Only one child in the RLMG group had grade 5 reflux on the affected side preoperatively, which remained as grade 3 reflux postoperatively. The RLMG and RLSPG groups experienced new reflux on the affected side after surgery, with the RLMG group having 1 case of grade 1 reflux and 2 cases of grade 3 reflux, while the RLSPG group had 4 cases of grade 3 reflux. No reflux was observed in the RLSG group based on the current follow-up results.

**Table 3:** Comparison of preoperative and postoperative renal hydronephrosis parameters.

Parameters	RLMG (*n* = 13)	RLSPG (*n* = 10)	RLSG (*n* = 7)	*P*
Follow-up (months)	12 (6.36)	7 (6.14)	6 (5.8)	<0.001
Follow-up Reflux level^a^				
I	1	0	0	0.229
II	0	0	0	
III	3	4	0	
Preoperative APD of renal pelvis (mm)	39.42 ± 13.64	22.37 ± 7.23	30.10 ± 13.71	0.008
Follow-up APD of renal pelvis (mm)	19.22 ± 7.51	11.39 ± 4.99	13.20 ± 4.34	0.013
Preoperative SFU grade^b^				
II	2	1	1	0.048
III	4	4	3	
IV	7	5	3	
Follow-up SFU grade				
I	5	6	3	0.477
II	8	4	3	
III	0	0	1	
Preoperative ureteral diameter (mm)	17.73 ± 4.44	20.10 ± 3.64	17.60 ± 3.06	0.295
Follow-up ureteral diameter (mm)	7.47 ± 3.68	6.79 ± 2.36	8.44 ± 2.28	0.544
Preoperative difference in DRF%	33.30 ± 24.30	30.72 ± 26.28	22.71 ± 13.72	0.629
Follow-up difference in DRF%	27.62 ± 21.59	28.65 ± 25.63	17.57 ± 12.90	0.532

APD, anteroposterior diameter; DRF, defferential renal function.

a According to the grading criteria of voiding cystourethrography (VCUG) used by the International Reflux Research Group in 1985.

b According to society for fetal urology (SFU)grading system.

The postoperative wound healing during the follow-up period of RLMG, RLSPG, and RLSG is shown in Figures 1D–F, respectively.

## Discussion

The refinement of intra-abdominal operations and minimally invasive surgical incisions are the development trends of pediatric minimally invasive surgery.Ureteral bladder reimplantation is the main method for treating lower ureteral stenosis. The traditional open ureteral bladder reimplantation surgery is considered the “gold standard” for this procedureWith the development of laparoscopic technology in the past decade, laparoscopic external ureteral reimplantation has been widely used due to its advantages of minimal trauma, fast postoperative recovery, and good cosmetic effects. Laparoscopic surgery for POM has become a routine treatment method in some centers (10). However, due to the long learning curve of laparoscopic surgery, especially the high requirements for laparoscopic techniques in the process of ureteral bladder anastomosis, the development of laparoscopic surgery has been limited. In 2,000, Da Vinci robots were approved for clinical use in the United States and were applied in the field of pediatric surgery the following year (11). However, due to limitations in instrument size, different disease types, a wide age range for children, and physiological and anatomical differences from adults, its development in the field of pediatric surgery has been relatively slow. In 2007, Uberoi et al. (12) first completed and reported robot assisted laparoscopic ureteral reimplantation surgery. Subsequently, there has been a continuous increase in relevant reports both domestically and internationally. The robot's 3D field of view and flexible instrument arm make the separation and cutting of the ureter more precise, and the suturing simpler. They can effectively avoid damage to the ureter and surrounding blood vessels, as well as avoid the angle and distortion of the ureter. Moreover, they have obvious advantages in establishing an external bladder muscle layer tunnel, suturing the bladder muscle layer and the urinary tract plasma muscle layer. These advantages are more prominent when fitting fine pipeline structures.Therefore, robot surgery can overcome the problem of long learning curve caused by laparoscopy and fully leverage its advantages in performing surgeries that require a large amount of suturing, especially in the anastomosis of pipelines (13, 14).

Since the introduction of robots in our department in 2020, we have applied robot assisted porous laparoscopic surgery to the treatment of common urinary system diseases in children, such as pyeloplasty (15). We found that the postoperative relief rate of hydronephrosis in the robot and laparoscopic groups was roughly the same, but the ratio of pyeloplasty time to total surgical time and postoperative hospital stay in the robot group were significantly lower than those in the laparoscopic group, and the amount of blood loss in the robot group was lower than that in the laparoscopic group, although the difference was not statistically significant.This is consistent with the findings of Edoardo et al. (16), who found that the robotic group had less surgical time than the laparoscopic group, and during follow-up, all patients were asymptomatic and had no recurrence of ureteropelvic junction obstruction (UPJO). Therefore, it is believed that the application of robots is as effective and safe as laparoscopy.

Robotic multi-ports surgery can significantly reduce the learning curve of the primary surgeon, making it easier to widely promote and apply. However, it also brings about problems such as scattered incisions and obvious scars after surgery. For pediatric patients, scars, even if small, will enlarge with growth and development. Therefore, it is particularly important to use minimally invasive surgery to bring postoperative aesthetics while treating diseases. For this reason, some scholars have imagined combining the da Vinci robot with a single-port laparoscope, that is, a robotic single-port laparoscope. However, due to the small body cavity space of children, single-port operation leads to more limited space, causing instrument crowding and collision, which once again increases the difficulty of surgery and reduces the advantages brought by robot operation. The Journal of Robotic Surgery has suggested against performing robotic surgery on infants, especially on low birth weight infants (17). Based on our extensive experience in single port laparoscopic surgery and robot operation in the early stage, while ensuring surgical safety and effectiveness, our team has increased the effective operating space inside the body cavity by adding another approximately 8 mm incision while retaining the minimally invasive concealed wound around the navel. This reduces the impact of chopstick effect, lowers the difficulty of single-port surgery, reduces the learning curve of the operator, and increases the scope of surgical application. At the same time, the wound is concentrated around the navel, concealed and aesthetically pleasing, improving the cosmetic effect of the wound. At present, this technology has been routinely applied in the treatment of common urinary tract and bile duct reconstruction surgeries in our department (8, 18, 19).

After accumulating sufficient experience in single port plus one robotic surgery, we gradually carried out single-port robotic surgery. Through our research, we found that although the surgery time was significantly longer in the RLSG and RLSPG than in the RLMG, there was no significant difference in the intra-abdominal ureteral reimplantation time among the three groups. The RLSG and RLSPG had a lower ratio of ureteral reimplantation time compared to the total surgery time. This is mainly due to the longer time required for trocar insertion and adjustment in the RLSG and RLSPG. With the accumulation of experience in single port operation, we believe that the surgery time in the single port group will gradually decrease in the future.Through follow-up, we also found that the postoperative recovery of the three groups, including the degree of renal pelvis and ureteral dilation, and the renal function on the affected side, had significantly improved compared to preoperative levels, and there was no significant difference among the three groups. In addition, technological advancements have led to an increase in the satisfaction ratings of patients' families with surgical wounds. The scores of RLSG group significantly higher than RLSG group and higher than RLSPG group, but the difference was not statistically significant.

It is worth mentioning that our current techniques have surpassed age limitations, with the minimum age for surgery being a 44-day-old child in the RLSG group. All three groups included children under 1 year old, and all had smooth postoperative recoveries.

This study has several limitations. Firstly, its retrospective design and relatively small sample size from a single center may introduce selection bias. Secondly, during the study period, no patients with bilateral POM were encountered, thus our findings are primarily applicable to unilateral cases. Future studies including bilateral POM are needed.

## Conclusion

In conclusion, robot-assisted laparoscopic ureteral reimplantation is a safe and effective treatment for pediatric POM. While the multi-ports approach offers the shortest operative time, the single-port technique provides superior cosmetic outcomes without compromising surgical efficacy or renal recovery. The single-port-plus-one represents a practical intermediate option. Surgeons can leverage these techniques to enhance cosmetic results and patient satisfaction following adequate training.

## Data Availability

The raw data supporting the conclusions of this article will be made available by the authors, without undue reservation.
